# Bone regeneration in rat calvarial defects using dissociated or spheroid mesenchymal stromal cells in scaffold-hydrogel constructs

**DOI:** 10.1186/s13287-021-02642-w

**Published:** 2021-11-14

**Authors:** Siddharth Shanbhag, Salwa Suliman, Samih Mohamed-Ahmed, Carina Kampleitner, Mohamed Nageeb Hassan, Patrick Heimel, Toni Dobsak, Stefan Tangl, Anne Isine Bolstad, Kamal Mustafa

**Affiliations:** 1 Center for Translational Oral Research (TOR), Department of Clinical Dentistry, Faculty of Medicine, University of Bergen, Årstadveien 19, 5009 Bergen, Norway; 2 Department of Immunology and Transfusion Medicine, Haukeland University Hospital, Bergen, Norway; 3 Core Facility Hard Tissue and Biomaterial Research/Karl Donath Laboratory, University Clinic of Dentistry, Medical University of Vienna, Vienna, Austria; 4 Ludwig Boltzmann Institute for Traumatology, The research center in cooperation with AUVA, Vienna, Austria; 5 Austrian Cluster for Tissue Regeneration, Vienna, Austria

**Keywords:** Xeno-free, Platelet lysate, MSC, Spheroid culture, Bone tissue engineering

## Abstract

**Background:**

Three-dimensional (3D) spheroid culture can promote the osteogenic differentiation of bone marrow mesenchymal stromal cells (BMSC). 3D printing offers the possibility to produce customized scaffolds for complex bone defects. The aim of this study was to compare the potential of human BMSC cultured as 2D monolayers or 3D spheroids encapsulated in constructs of 3D-printed poly-L-lactide-co-trimethylene carbonate scaffolds and modified human platelet lysate hydrogels (PLATMC-HPLG) for bone regeneration.

**Methods:**

PLATMC-HPLG constructs with 2D or 3D BMSC were assessed for osteogenic differentiation based on gene expression and in vitro mineralization. Subsequently, PLATMC-HPLG constructs with 2D or 3D BMSC were implanted in rat calvarial defects for 12 weeks; cell-free constructs served as controls. Bone regeneration was assessed via in vivo computed tomography (CT), ex vivo micro-CT and histology.

**Results:**

Osteogenic gene expression was significantly enhanced in 3D versus 2D BMSC prior to, but not after, encapsulation in PLATMC-HPLG constructs. A trend for greater in vitro mineralization was observed in constructs with 3D versus 2D BMSC (*p* > 0.05). In vivo CT revealed comparable bone formation after 4, 8 and 12 weeks in all groups. After 12 weeks, micro-CT revealed substantial regeneration in 2D BMSC (62.47 ± 19.46%), 3D BMSC (51.01 ± 24.43%) and cell-free PLATMC-HPLG constructs (43.20 ± 30.09%) (*p* > 0.05). A similar trend was observed in the histological analysis.

**Conclusion:**

Despite a trend for superior in vitro mineralization, constructs with 3D and 2D BMSC performed similarly in vivo. Regardless of monolayer or spheroid cell culture, PLATMC-HPLG constructs represent promising scaffolds for bone tissue engineering applications.

**Supplementary Information:**

The online version contains supplementary material available at 10.1186/s13287-021-02642-w.

## Background

Reconstruction of advanced maxillofacial bone deficiencies is a clinical challenge. Bone tissue engineering (BTE) strategies are increasingly being used to overcome the limitations of autogenous bone grafts and existing bone-substitute materials to reconstruct such defects [1]. BTE aims to combine the cellular (*osteogenic* cells), extracellular (*osteoconductive* scaffolds) and/or molecular elements (*osteoinductive* growth factors) required for bone healing [2]. The potential of BTE for orofacial bone regeneration as demonstrated in several preclinical and clinical studies has recently been summarized [1, 3–5].

BTE strategies usually involve the use of adult mesenchymal stromal cells (MSC), most frequently derived from the bone marrow (BMSC) and expanded as plastic-adherent monolayers [6, 7]. This expansion process can be further enhanced by replacing animal-derived supplements, e.g., fetal bovine serum (FBS), in MSC cultures with humanized or “xeno-free” alternatives such as human platelet lysate (HPL) [8–10]. This step is important not only to enhance the efficacy of MSC expansion but also to facilitate clinical translation of cell therapies according to current regulations [11]. Despite these advances, the two-dimensional (2D) monolayer expansion system is not representative of the in vivo MSC microenvironment and may alter the phenotype and properties of MSC [12, 13]. In contrast, self-assembly or aggregation of MSC into three-dimensional (3D) spheroids simulates more closely their in vivo microenvironment or niche [12, 14]. In the context of bone regeneration, the cytoskeletal changes induced by 3D culture may be particularly beneficial [15, 16]. We have recently reported that 3D spheroid culture of BMSC promotes the expression of several genes and proteins associated with self-renewal and osteogenic differentiation; the latter is independent of osteogenic stimulation [17]. Moreover, several studies have demonstrated benefits of spheroid culture for promoting the differentiation [18–20], paracrine function [21] and regeneration potential of MSC [22–25].

Traditional cell delivery methods involve direct seeding and attachment of MSC on biomaterial scaffolds before in vivo transplantation [26]. However, this method may not be optimal for the delivery of cell spheroids where the 3D structure, essential to maximize their in vivo effects, is lost by direct seeding. To preserve the 3D structure, encapsulation of spheroids in hydrogels represents an effective delivery system for BTE applications [27–29]. Recent reports also suggest that hydrogel properties may modulate the efficacy of MSC spheroids [30]. Since HPL is increasingly being used, and even recommended, for clinical-grade MSC culture [31], extending its application as a hydrogel carrier represents a clinically relevant and cost-effective strategy for BTE. In addition to functioning as cell carriers, HPL hydrogels (HPLG) may offer an additional benefit of sustained cytokine release at regeneration sites [32].

While hydrogel scaffolds may be used in self-contained bone defects, larger, non-contained defects often necessitate the use of rigid biomaterials. These “bone substitute” biomaterials represent the cornerstone of bone regenerative therapies, and various materials have been investigated to date [33]. Among these are synthetic polymers, e.g., poly(L-lactic acid) (PLA), poly(glycolic acid) (PGA), and their *co*polymers, e.g., polylactic-co-glycolic acid (PLGA). A major advantage of synthetic (co)polymers is the possibility to adjust their structure, biomechanical properties and biodegradability to suit the required application(s), in addition to a reduced risk of undesirable immunological reactions (34, 35). Moreover, current advances in 3D printing allow the fabrication of customized (co)polymer scaffolds with highly controlled macro- and micro-architecture for bone regeneration [36]. Although PLA, PGA and PLGA represent some of the most frequently used materials for 3D-printed bone scaffolds, a major disadvantage is the local pH alterations caused by the acidic by-products from their hydrolytic degradation, which may be unfavorable for cell growth and differentiation [35]. Trimethylene carbonate (TMC) is a polymer which degrades via surface erosion; when combined with PLA (PLATMC), it stabilizes the PLA resulting in less hydrolysis and thereby less by-products and local pH alterations [35]. The suitability of PLATMC for producing 3D-printed scaffolds, which support MSC attachment, growth and differentiation, has recently been demonstrated [37].

A combination of MSC with growth factor-rich hydrogels (HPLG) and biomaterial scaffolds (PLATMC), reflecting the classical tissue engineering “triad,” may represent a novel and effective strategy for bone regeneration in challenging defects [38, 39]. Therefore, the objectives of the present study were to develop constructs of BMSC encapsulated in HPLG and PLATMC constructs as dissociated (2D) cells or 3D spheroids and to compare their in vivo bone regeneration potential in an orthotopic defect model.

## Methods

### Cell culture

The use of human cells and tissues was approved by the Regional Committees for Medical Research Ethics (REK) in Norway (2013-1248/REK sør-øst C). Bone marrow aspirates were obtained from three donors (1 female and 2 males; 8–10 years) undergoing corrective surgery at the Department of Plastic Surgery, Haukeland University Hospital, Bergen, Norway. BMSC were isolated and expanded in growth media (GM) comprising of Dulbecco’s modified Eagle’s medium (DMEM, Invitrogen, Carlsbad, CA, USA) supplemented with 5% (v/v) pooled HPL (Bergenlys, Bergen, Norway), 1% (v/v) penicillin/streptomycin (GE Healthcare, South Logan, UT, USA) and 1 IU/mL heparin (Leo Pharma AS, Lysaker, Norway). The preparation of HPL is described elsewhere [10]. Cells were sub-cultured (4000 cells/cm^2^) and expanded in humidified 5% CO_2_ at 37 °C; passage 2–4 cells were used in experiments. Monolayer (2D) BMSC were characterized based on immunophenotype, proliferation and multi-lineage differentiation potential as previously described [10].

To generate 3D spheroids, monolayer BMSC (passage 2) were seeded in microwell-patterned 24-well plates (Kugelmeiers Ltd, Erlenbach, Switzerland) in GM; after 24 h, aggregates of ~ 1000 cells were formed via guided self-assembly [17]. To induce differentiation of 2D and 3D BMSC, osteogenic induction media (OIM) were prepared by supplementing GM with final concentrations of 0.05 mM L-ascorbic acid 2-phosphate, 10 nM dexamethasone and 10 mM β glycerophosphate (all from Sigma-Aldrich, St. Louis, MO, USA).

### Characterization of 2D and 3D BMSC

Monolayer (2D) and spheroid (3D) BMSC were characterized at gene and protein levels. Expressions of genes associated with multipotency and osteogenesis (Additional file 1: Table 1), normalized to that of glyceraldehyde 3-phosphate dehydrogenase (GAPDH), were assessed after 7 days via quantitative real-time polymerase chain reaction (qPCR) using TaqMan PCR assays (Thermo Scientific, Carlsbad, CA, USA). Osteogenic gene expression was assessed in both GM and OIM cultures. RNA extraction and cDNA synthesis were performed as previously described [40]. Mineralization in 2D and 3D BMSC was confirmed via Alizarin red S staining (Sigma-Aldrich) after 21 days of OIM culture.

For protein-level characterization, conditioned media (CM) from 2D (2D-CM) and 3D BMSC (3D-CM) were collected after culturing the cells for 48 h in HPL-free media and characterized via a multiplex cytokine assay as previously described [17]. Briefly, the concentrations of 15 cytokines (Additional file 1: Table 2) were measured using a custom multiplex assay and Bio-Plex R 200 System (both from Bio-Rad Laboratories, CA, USA), according to the manufacturer’s instructions. To account for differences in cell proliferation rates between 2 and 3D cultures, cytokine concentrations (pg/mL) were normalized to the corresponding total cellular DNA (ng/mL). DNA quantification was performed using the Quant-IT PicoGreen dsDNA Assay (Invitrogen) according to the manufacturer’s instructions. The efficacy of 2D- and 3D-CM was tested in an in vitro wound healing assay of rat BMSC (Additional file 1).

### 3D printing of PLATMC scaffolds

3D-printed PLATMC scaffolds were produced as described elsewhere [37]. Briefly, a 3D CAD model was designed using the Magics software integrated with a 3D-Bioplotter (both from EnvisionTEC, Gladbeck, Germany). Granules of medical-grade PLATMC (RESOMER® LT-706-S 70:30, Evonik GmBh, Essen, Germany) were loaded into the printer cartridge (pre-heated to 220 °C), and rectangular sheets of three layers with an orientation of 0°–90°–0° were printed at 190 °C with an inner nozzle diameter of 400 μm and strand spacing of 0.7 mm [37]. Disc-shaped scaffolds measuring 5 mm × 1.2 mm were punched out and placed in 48-well plates. Prior to use in experiments, the scaffolds were sterilized by soaking in 70% ethanol for 30 min, followed by washing with phosphate-buffered saline (PBS; Invitrogen) and 2-h exposure to UV light in sterile conditions.

### Production of hydrogels and constructs

HPLG were produced by combining previously reported methods for platelet-rich plasma (PRP) and fibrin gel preparation, both of which are commonly used as scaffolds in BTE applications. To prepare the hydrogels, sterile-filtered HPL (same as for cell culture) was supplemented with 20 mg/mL fibrinogen (Sigma-Aldrich) to increase the stiffness and mechanical properties of the hydrogel. Gelation was achieved by adding a “thrombin solution” containing 1 IU/mL human thrombin and 1 TIU/mL aprotinin in 20 mM CaCl_2_ solution (all from Sigma-Aldrich), followed by incubation at 37 °C for 15 min.

To prepare the PLATMC-HPLG constructs, HPL/fibrinogen and thrombin solutions were mixed and 50 μL were quickly seeded on the PLATMC scaffolds (pre-wetted with HPL), followed by incubation at 37 °C for 15 min. Imaging of constructs was performed using a stereomicroscope (Leica M205C, Heerbrugg, Switzerland) and, after gold/palladium sputter-coating, using a scanning electron microscope (SEM; Phenom XL, Thermo Scientific).

### Cell encapsulation in constructs

For cell encapsulation, equal numbers of dissociated (2D) or spheroid (3D) BMSC were uniformly suspended in fibrin-supplemented HPL, mixed with thrombin solution and seeded on scaffolds (1 × 10^6^ cells in 50 μL) as described above. The distribution of 2D and 3D BMSC within PLATMC-HPLG constructs was observed under a light microscope (Nikon Eclipse TS100, Tokyo, Japan). Cell morphology and viability were assessed after 1, 7 and 21 days using the LIVE/DEAD cell viability assay (Invitrogen) and observed under a high-speed Andor Dragonfly 5050 confocal microscope equipped with an iXon 888 Life EMCCD camera (1024 × 1024 resolution, 100–200 × magnification; Oxford Instruments, Abingdon, UK). Z-stacks were acquired from the top of each construct, with steps of 4 μm to a depth of up to 200 μm. Images were processed using the Imaris software (Oxford Instruments).

To assess osteogenic differentiation, PLATMC-HPLG constructs with 2D or 3D BMSC were cultured in GM and OIM for up to 21 days. Expressions of early, intermediate and late osteogenesis-related genes (Additional file 1: Table 1) were assessed after 7 days via qPCR. In vitro mineralization was assessed via Alizarin red S staining (Sigma-Aldrich) after 14 and 21 days, as previously described [40]. For quantification, the stain was dissolved in 10% cetylpyridinium chloride (Sigma-Aldrich) and absorbance was measured at 540 nm using a microplate reader.

### Implantation in rat calvarial defects

Animal experiments were approved by the Norwegian Animal Research Authority (Mattilsynet; FOTS-17443) and performed in accordance with the ARRIVE guidelines [41]. Twelve male athymic nude rats (Rj:ATHYM-Foxn1rnu, Janvier Labs, Le Genest-Saint-Isle, France), 7 weeks old and weighing 300 ± 15.58 g were used. Animals were housed in stable conditions (22 ± 2 °C) with a 12-h dark/light cycle and ad libitum access to food and water. Animals were allowed to acclimatize for one week prior to experiments and were regularly monitored for signs of pain/infection, food intake and activity during the entire experimental period. Pre-operatively, animals were anesthetized with a mixture of sevoflurane (Abbott Laboratories, Berkshire, UK) and O_2_ using a custom-made mask. Following anesthesia, a 2-cm sagittal incision was made in the midline of the cranium to reflect the periosteum and expose the parietal bones. In each animal, two full-thickness defects of 5 mm diameter [42] were created on either side using a trephine bur (Meisinger GmbH, Neuss, Germany) attached to a slow-speed handpiece under saline irrigation. Special care was taken to preserve the sagittal suture and underlying dura mater. The following constructs were then randomly implanted in the defects: PLATMC-HPLG containing 2 × 10^6^ 2D BMSC (*n* = 8), PLATMC-HPLG containing 2 × 10^6^ 3D BMSC (*n* = 8) or cell-free PLATMC-HPLG constructs (*n* = 6); PLATMC scaffolds without HPLG were implanted in two defects (*n* = 2). The critical-size nature of 5 mm defects was previously tested showing no healing within the observation time (data not shown). All constructs were cultured in GM for 36 h prior to implantation. Randomization was performed so that no animal received two constructs from the same group and animals were coded via ear clips. Post-operatively, the skin was sutured (Vicryl, Ethicon, Somerville, NJ, USA) and animals were injected subcutaneously with buprenorphine (Temgesic 0.03 mg/kg, Schering-Plough, UK) for up to 2 days thereafter. After 12 weeks, the animals were euthanized with an overdose of CO_2_. The primary outcome was assessment of bone regeneration in the defects via radiography and histology. For all subsequent handling/analyses, the animals were identified by numbers to facilitate blinding of observers to the treatment groups.

### In vivo* computed tomography (CT)*

To track in vivo bone regeneration, the calvaria were scanned 4, 6, 8 and 12 weeks after surgery using a small-animal CT scanner (nanoScan, Mediso, Budapest, Hungary) as previously described [43]. At each time point, 0.04 mm resolution scans were obtained and analyzed using PMOD software (PMOD Technologies LLC, Zurich, Switzerland). A standardized volume of interest (VOI)—including the entire thickness of the defect and excluding 0.5 mm of marginal bone, was defined for each defect. A density threshold was applied to exclude the scaffold (determined by scanning blank scaffolds using the same parameters) and classify only mineralized tissues. Percentage defect fills in the VOI, i.e., new bone volume per total defect volume (nBV/TV), were calculated using the PMOD software.

### Ex vivo* micro-CT and histology*

Immediately after euthanasia, the calvaria were harvested and fixed in 10% buffered formalin. For micro-CT (μCT) analysis, specimens were scanned using a SCANCO 50 μCT scanner (SCANCO Medical AG, Bruttisellen, Switzerland) at 90 kV and 200 μA with an isotropic resolution of 17.2 μm. Scans were reconstructed by orienting the drill direction along the Z-axis, with the defect in the approximate center of the image, using Amira software (Thermo Scientific). A standardized VOI (as described for in vivo CT) and threshold were applied to all samples. In addition to nBV/TV (as described for the CT), the formation of bone “islands” or isolated areas of new bone not connected to the host bone [isolated bone volume per total defect volume (iBV/TV)], was calculated using ImageJ software [44].

After μCT scanning, the calvaria specimens were processed for undecalcified histology. Specimens were dehydrated in ascending grades of alcohol and embedded in light-curing resin (Technovit 7200 + 1% benzoyl peroxide, Kulzer & Co., Wehrheim, Germany). Blocks were further processed using EXAKT cutting and grinding equipment (EXAKT Apparatebau, Norderstedt, Germany). Standardized thin-ground sections (~ 100 μm) parallel to the sagittal suture and perpendicular to the parietal bone (Additional file 1: Figure 1), were prepared from all specimens and stained with Levi-Laczko dye (Morphisto GmbH, Frankfurt, Germany). In this staining, mature bone appears light pink, woven bone appears dark pink, and soft tissue (including collagen) appears dark blue [45]. Further, the sections were scanned using an Olympus BX61VS digital virtual microscopy system (DotSlide 2.4, Olympus, Tokyo, Japan) with a 20 × objective resulting in a resolution of 0.32 µm per pixel.

For histomorphometric analysis, a standardized region of interest (ROI) was defined within each defect excluding 1 mm of marginal bone. Using Definiens Developer XD2 software (Definiens, Munich, Germany), the different tissue types (bone/soft tissue/scaffold) were semi-automatically classified from digital images and further corrected using Adobe Photoshop software (Adobe, San Jose, CA, USA). The percentage of new bone formation in the ROI was calculated as a ratio of the area of newly formed bone to the total available area (nB.Ar/T.Ar). Blood vessels, identified by endothelial lining and entrapped erythrocytes, were manually counted in the ROI.

### Statistical analysis

Statistical analysis was performed using the Prism 9 software (GraphPad Software, San Diego, CA, USA). Data are presented as means (± SD and/or range), unless specified. Analyses of gene expression data are based on delta-CT values, and results are presented as relative (log/nonlinear) fold changes in 3D versus 2D BMSC using scatter plots. Multiplex proteomic data are presented on a logarithmic (log_10_) scale. All other linear data are presented as bar graphs. Normality testing was performed via the Shapiro–Wilk test. The Student's *t* test, Mann–Whitney U test, one-way analysis of variance (ANOVA; followed by a post hoc Tukey’s test) or Kruskal–Wallis test, were applied as appropriate, and *p* < 0.05 was considered statistically significant.

## Results

### Gene expression and cytokine secretion are altered in spheroid BMSC

**Fig. 1:**
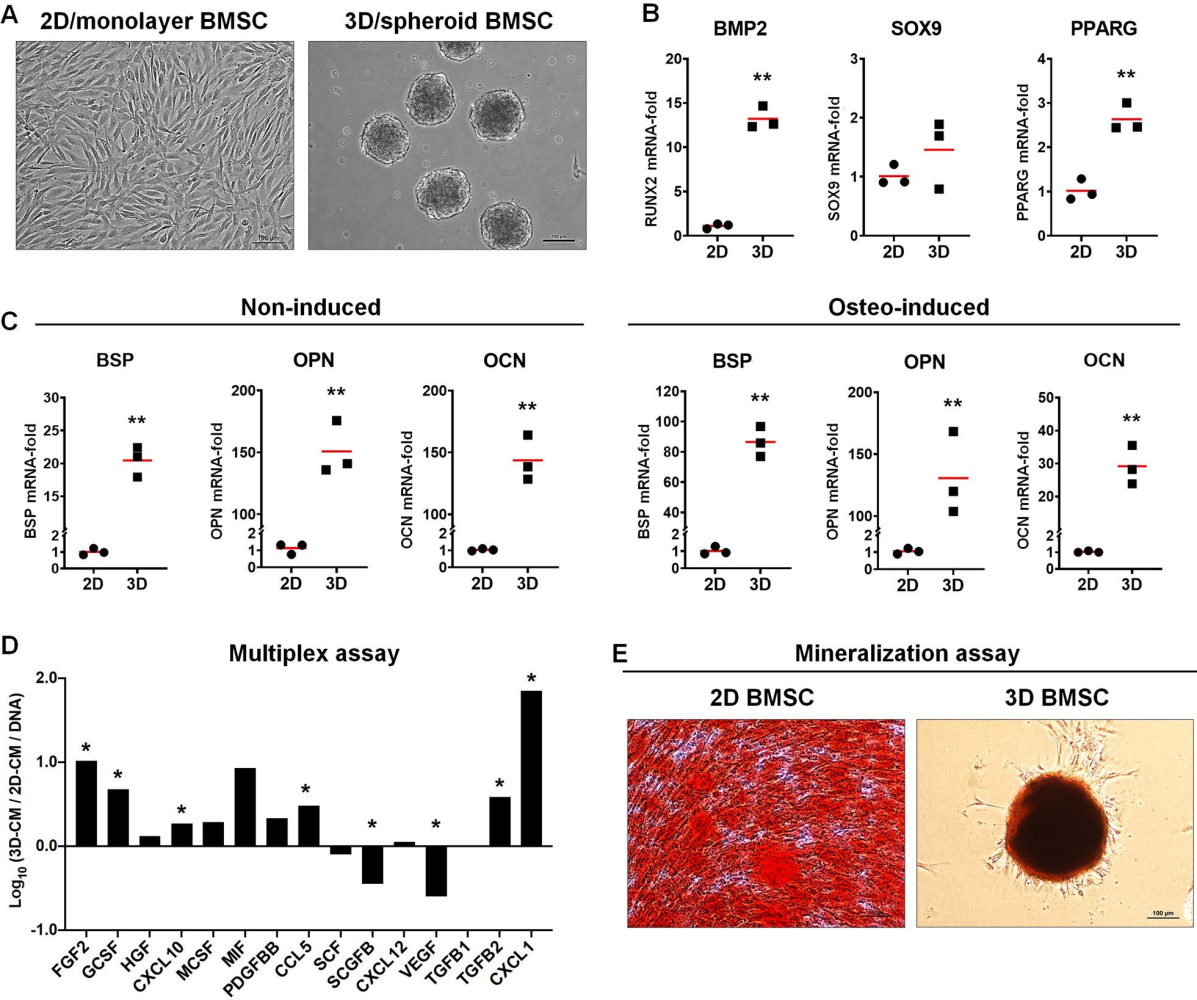
Characterization of 2D and 3D BMSC. **a** Representative images of 2D and 3D BMSC, scale bars 100 µm. Expression (fold changes) of multipotency-related genes (**b**) and osteogenesis-related genes (**c**) in 3D BMSC relative to 2D BMSC after 7 days; the latter were assessed in growth (non-induced) or induction media (osteo-induced). Data represent means of 3 experimental replicates; statistical analyses are based on delta-Ct values; *p < 0.05; **p < 0.001. **d** Protein (cytokine) concentrations (pg/mL) in the conditioned media (CM) of 2D and 3D BMSC normalized to corresponding DNA contents (ng/mL); data are presented as the logarithm (log10) of the ratio of means of each cytokine in 3D-CM/2D-CM; *p < 0.05. **e** Mineralization in 2D and 3D BMSC detected via Alizarin red staining, scale bars 100 µm. BMP2, bone morphogenetic protein 2; SOX9, sex determining region Y-box 9; PPARG, peroxisome proliferator-activated receptor gamma; BSP, bone sialoprotein; OPN/SPP1, osteopontin; OCN/BGLAP osteocalcin. Multiplex assay (see Additional file 1: Table 2)

Monolayer BMSC showing characteristic morphology, immunophenotype and multi-lineage differentiation potential were expanded in HPL supplemented GM (Additional file 1: Figure 2); passage 2 cells were used to form 3D spheroids as previously described [17] (Fig. 1a). After 7 days, significant upregulations of genes associated with early osteogenic [bone morphogenetic protein 2 (BMP2), 13.20-fold, *p* = 0.0001] and adipogenic differentiation [peroxisome proliferator-activated receptor gamma (PPARG), 2.63-fold, *p* = 0.0028] were observed in 3D versus 2D BMSC; upregulation of chondrogenic differentiation gene SRY-box transcription factor 9 (SOX9) was not statistically significant (1.45-fold, *p* > 0.05) (Fig. 1b). Genes for extracellular matrix (ECM) components associated with late-stage osteogenic differentiation, i.e., bone sialoprotein (BSP; 20.45-fold, *p* < 0.0001), osteocalcin (OCN/BGLAP; 150.83-fold, *p* < 0.0001) and osteopontin (OPN/SPP1; 143.73-fold, *p* < 0.0001), were also upregulated in 3D versus 2D BMSC, regardless of osteogenic induction (Fig. 1c). In vitro mineralization was confirmed after 21 days of induction in both 2D and 3D BMSC (Fig. 1e).

The concentrations of various growth factors and chemokines were measured in 2D- and 3D-CM. Several growth factors were elevated in 3D- versus 2D-CM: fibroblast growth factor (FGF2; *p* < 0.05), hepatocyte growth factor (HGF; *p* > 0.05), granulocyte colony-stimulating factor (GCSF; *p* < 0.05), platelet-derived growth factor (PDGF-BB; *p* > 0.05) and transforming growth factor beta (TGF-β2; *p* < 0.05). Chemokine ligands 1 (CXCL1/GROα; *p* < 0.05), 10 (CXCL10; *p* < 0.05) and 5 (CCL5; *p* < 0.05) were also elevated in 3D-CM. Stem cell factor (SCF; *p* > 0.05), vascular endothelial growth factor (VEGF; *p* < 0.05) and stem cell growth factor beta (SCGF-β; *p* < 0.05) were greater in 2D-CM (Fig. 1d). Comparable in vitro wound closure was observed in rat BMSC exposed to 2D- or 3D-CM for 24–48 h (*p* < 0.05; Additional file 1: Figure 3).

### PLATMC-HPLG constructs maintain the activity of 2D and 3D BMSC in vitro

**Fig. 2:**
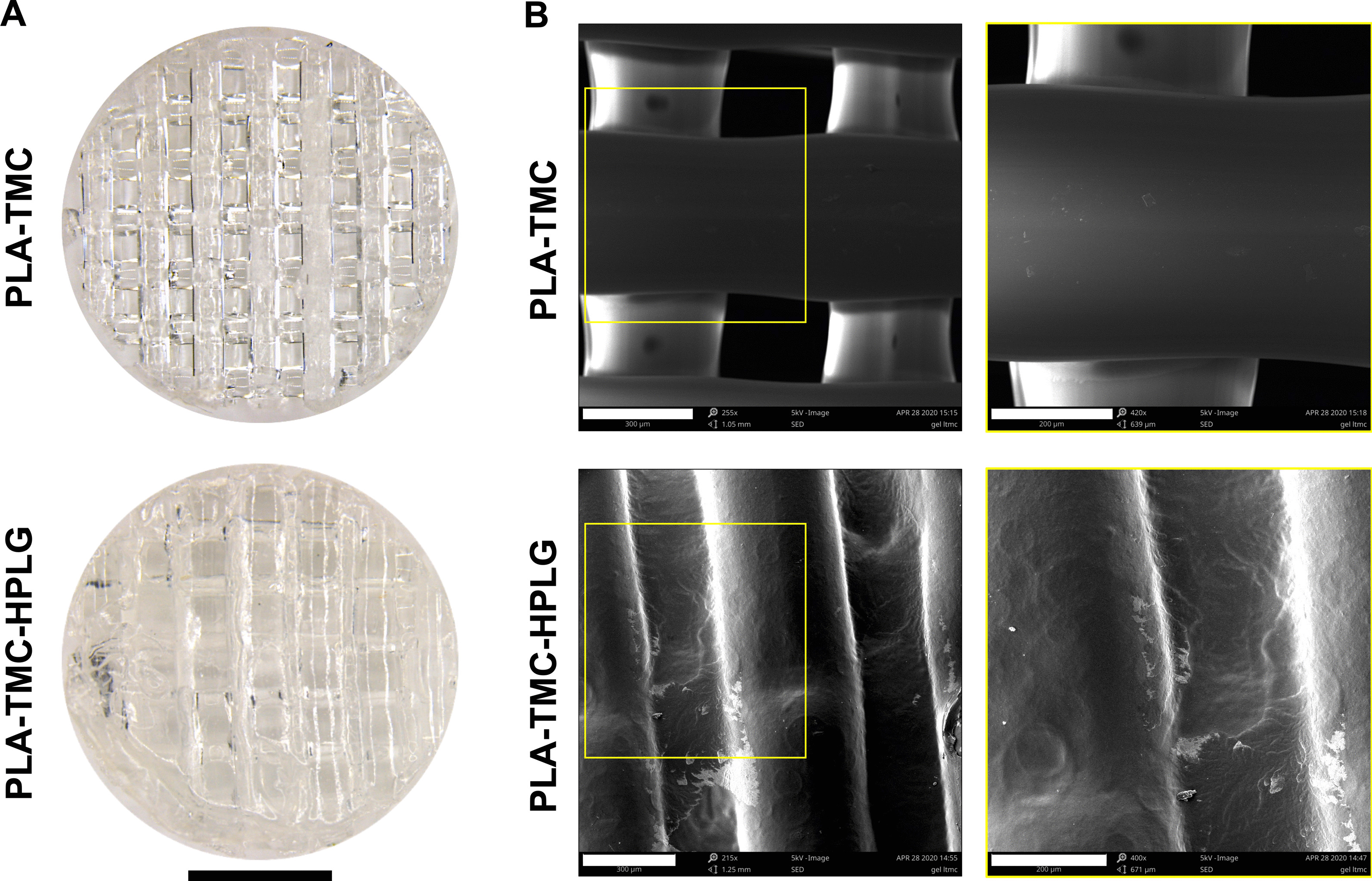
Scaffold-hydrogel constructs. **a** Stereomicroscopic images of PLATMC scaffolds before (top) and after encapsulation in HPLG (bottom), scale bar 2 mm. **b** Corresponding SEM images of PLATMC and PLATMC-HPLG constructs in low (left; scale bars 300 µm) and high magnification (right; scale bars 200 µm)

PLATMC scaffolds were 3D-printed with a pore size of 350–400 μm and total porosity of 53.96% ± 2.91% as determined by µCT. Modified HPLG were prepared by addition of fibrinogen and thrombin. When combined with HPLG, the scaffold filaments and pores were completely covered, indicating the potential for high “cell-seeding efficacy” (Fig. 2a, b).

**Fig. 3:**
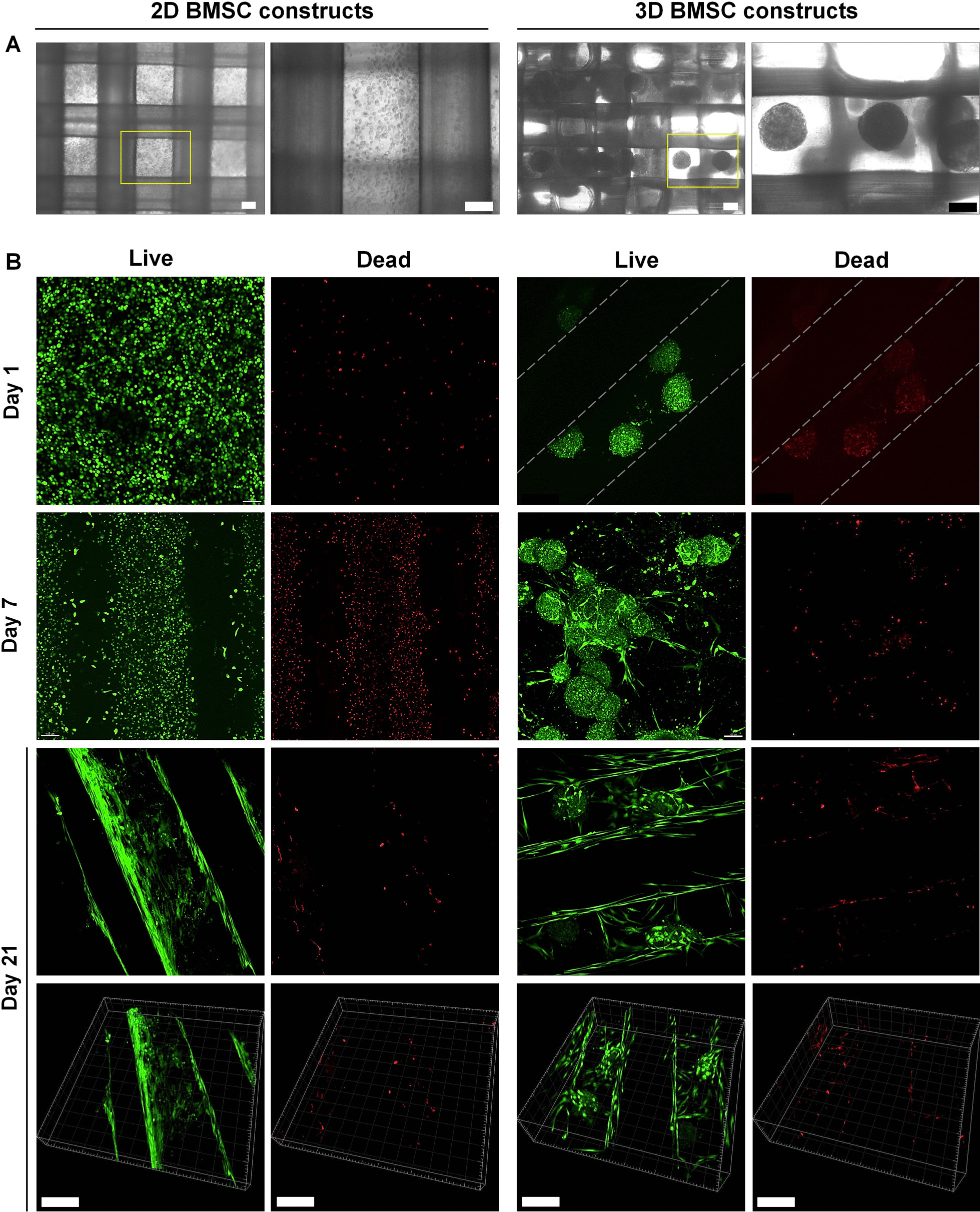
Cell seeding in scaffold-hydrogel constructs. **a** Distribution of 2D and 3D BMSC in constructs after seeding, scale bars 100 µm. **c** Representative confocal images showing cell viability based on the live (green) and dead (red) assay after 1, 7 and 21 days; corresponding 3D z-stack views of constructs at 21 days showing cell spreading on and/or in between the scaffold filaments; dotted lines indicate outlines of the printed filaments; scale bars 200 µm

Constructs containing equal numbers of 2D or 3D BMSC were produced; uniform distribution of cells/spheroids was confirmed soon after encapsulation (Fig. 3a). After 24 h, both single and spheroid BMSC appeared rounded and suspended mainly within the gels and not directly attaching to the scaffold surface (Fig. 3b). After 7 days, proliferation and spreading of cells within the hydrogels was observed, with a tendency for more dead cells in 2D versus 3D BMSC constructs. In the case of 3D BMSC, the spheroid structure appeared to still be maintained, although several cells appeared to migrate from the spheroids into the gel. After 21 days, the hydrogel was substantially degraded and 2D BMSC appeared to attach and spread on the surface of the PLATMC filaments. In 3D BMSC, the spheroid structure was still preserved after 21 days, and, in contrast to 2D BMSC, the cells appeared to spread both on the PLATMC filaments and in the spaces in between (Fig. 3b).

**Fig. 4:**
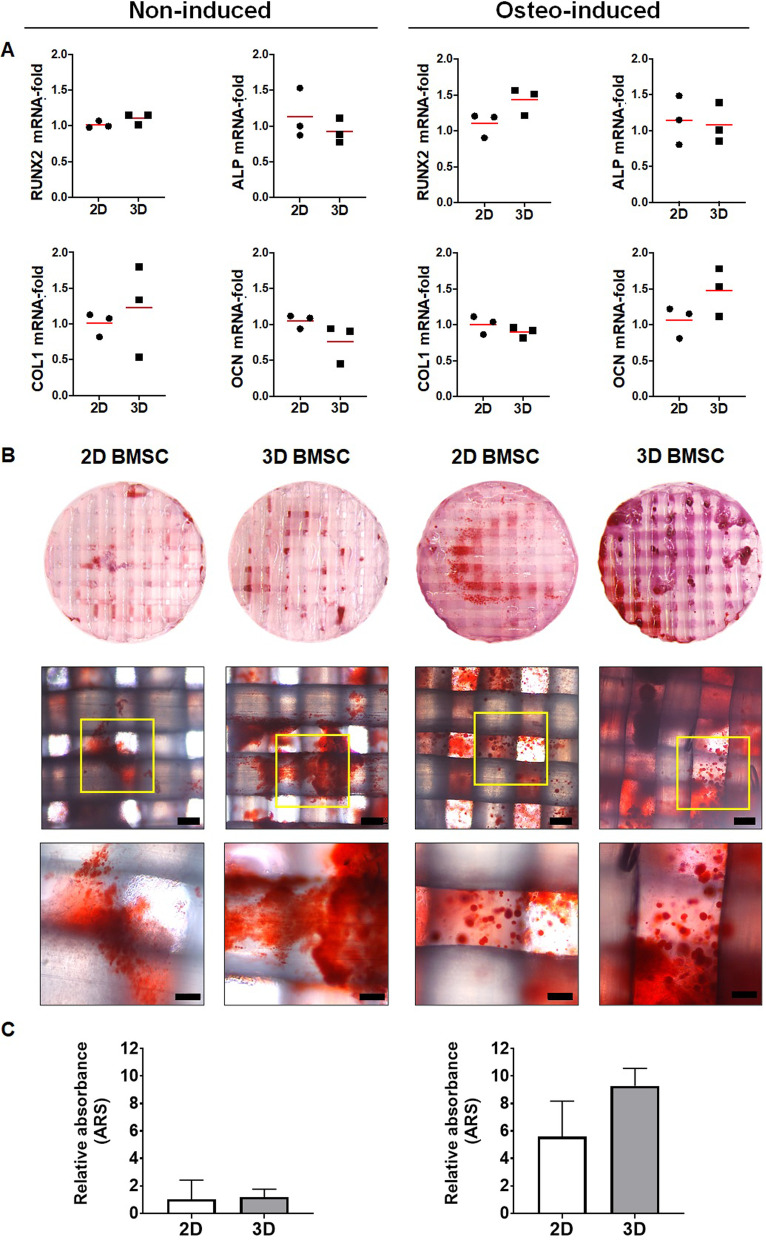
In vitro osteogenic differentiation. **a** mRNA fold changes of osteogenesis-related genes in 3D-relative to 2D-BMSC constructs after non-induced or osteogenic (osteo-induced) culture for 7 days. Data represent means (*n* = 3); no significant differences in 3D versus 2D BMSC for any of the genes. **b** Representative macroscopic and corresponding microscopic images of in vitro mineralization (Alizarin red S) in 2D and 3D BMSC constructs after non-induced or osteo-induced culture for 21 days, scale bars 100 µm. **c** Quantification of Alizarin red staining via absorbance measurements in non-induced and induced 2D and 3D BMSC constructs; data represent means and SD (*n* = 3 or 4) of absorbance values relative to the non-induced 2D BMSC group. RUNX2 runt-related transcription factor 2; ALPL alkaline phosphatase, COL1A2, collagen type 1-alpha 2; OCN/BGLAP, osteocalcin

Gene expression analysis of encapsulated 2D and 3D BMSC revealed no significant changes in early [runt-related transcription factor 2 (RUNX2)], intermediate [alkaline phosphatase (ALP), collagen type 1 (COL1)] or late (OCN) osteogenic differentiation markers after 7 days, regardless of induction (Fig. 4a); a trend for upregulation of RUNX2 (1.43-fold) and OCN (1.47-fold) was observed in induced 3D versus 2D BMSC (*p* > 0.05). Alizarin red staining revealed comparable in vitro mineralization in 2D versus 3D BMSC constructs after 14 days in OIM. After 21 days, a trend for greater mineralization was observed in 3D versus 2D BMSC constructs in OIM (*p* > 0.05; Fig. 4b, c). Evidence of mineralization was also observed in non-induced constructs of 2D and 3D BMSC, although significantly lower than in the corresponding induced constructs (*p* < 0.05).

### *Comparable bone regeneration in PLATMC-HPLG constructs with 2D or 3D BMSC *in vivo

**Fig. 5:**
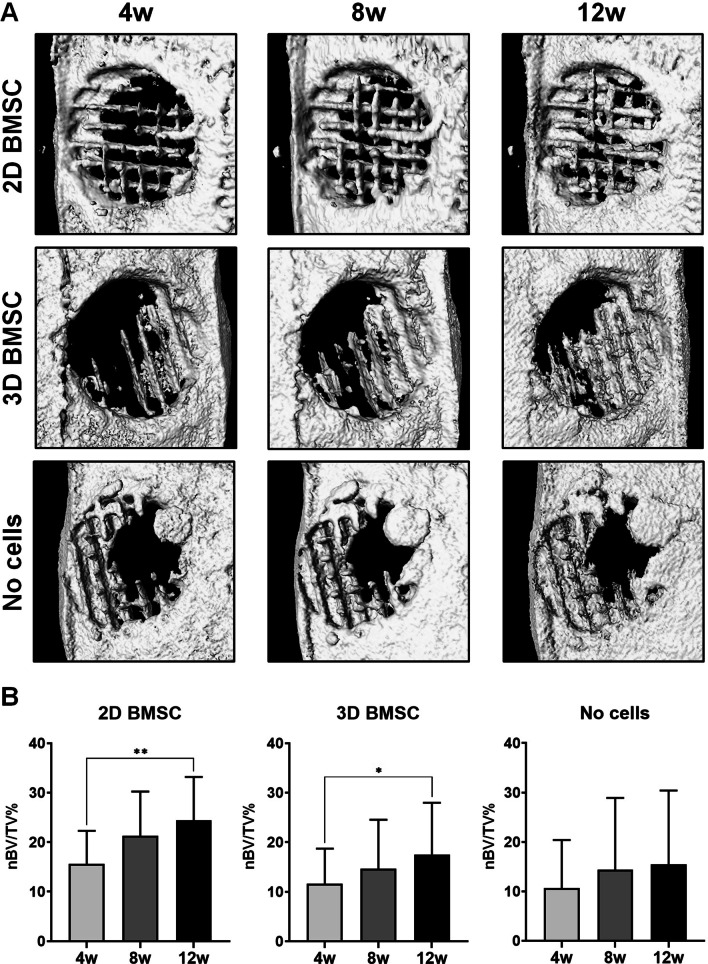
In vivo CT scanning. Representative reconstructed CT scans showing progression and distribution of bone regeneration (**a**) and corresponding quantification (**b**) in constructs with 2D BMSC, 3D BMSC and no cells from 4 to 12 weeks (w); data represent means ± SD; **p < 0.01; *p < 0.05

All experimental animals recovered from surgery and no adverse events were observed. In vivo CT scanning revealed bone regeneration of varying degrees in all defects after 4 weeks, increasing progressively up to 12 weeks, in all groups, i.e., PLATMC-HPLG constructs with 2D BMSC, 3D BMSC or no cells (Fig. 5a). In constructs with 2D BMSC and 3D BMSC, the increase in bone formation from 4 to 12 weeks was statistically significant (*p* < 0.05; Fig. 5b). Bone formation typically started from the defect margins and progressed towards the center, closely following the structure of the scaffolds, i.e., in the pores and along the printed filaments. Islands of new bone, not connected to the host bone, were also observed. Although a trend for greater bone formation was observed in constructs with 2D BMSC, no significant differences were observed between the groups at 4 (*p* = 0.437), 8 (*p* = 0.355) or 12 weeks (*p* = 0.383).

**Fig. 6:**
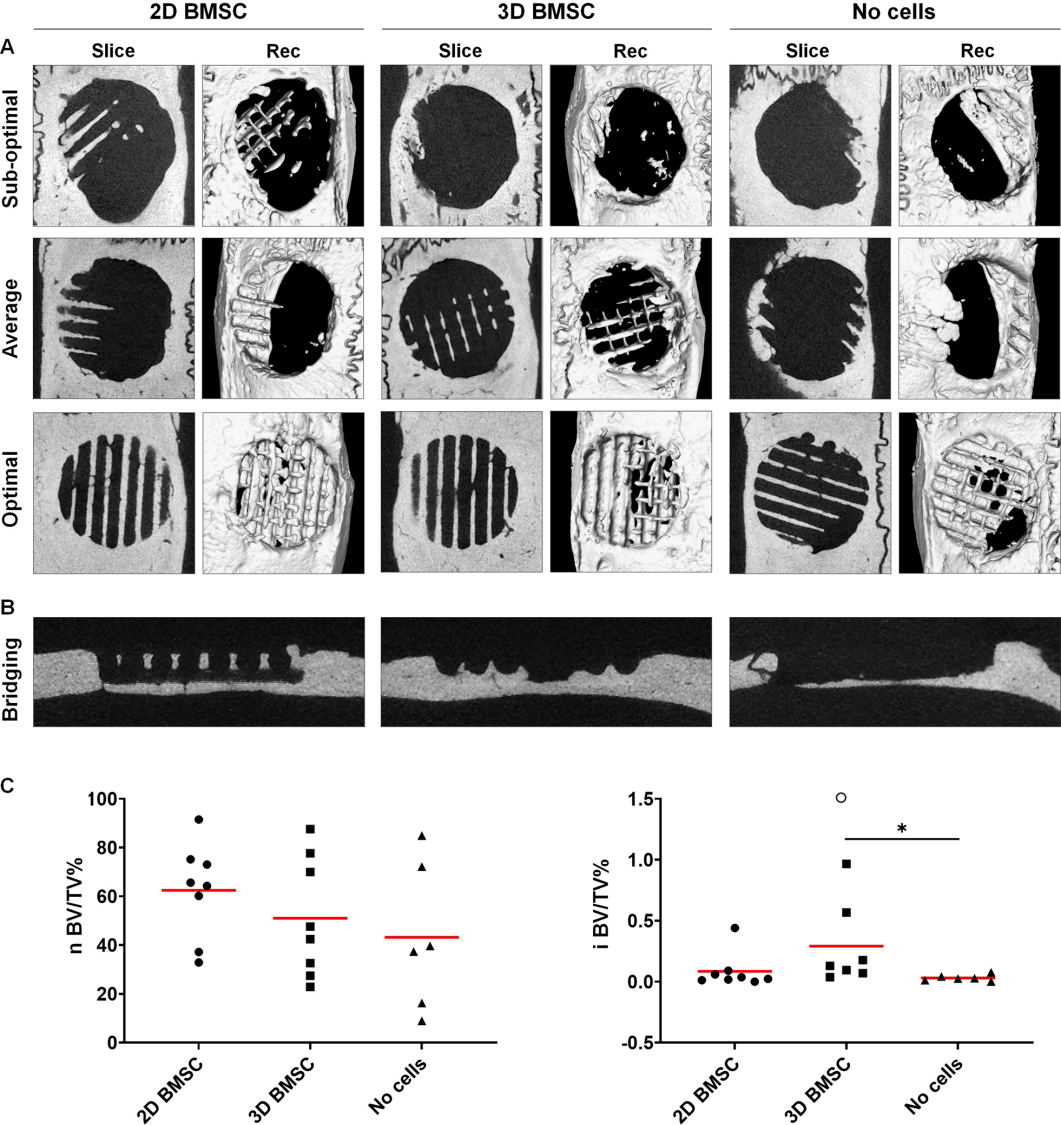
Micro-CT analysis. **a** Representative slice and 3D reconstructed (Rec) images of sub-optimal, average and optimal bone regeneration (based on quantitative analysis) in constructs with 2D BMSC, 3D BMSC and no cells after 12 weeks. **b** Representative images of defect bridging. **c** Quantification of total bone (nBV/TV%) and island bone regeneration (iBV/TV%); o indicates an outlier value (iBV/TV 1.70%); data represent means, *p < 0.05

The in vivo CT findings were confirmed by ex vivo μCT after 12 weeks (Fig. 6a). Central slices revealed bone formation throughout the entire thickness of the defects with complete bridging, i.e., transverse defect closure, in 75%, 62.5% and 33.3% of constructs with 2D BMSC, 3D BMSC and no cells, respectively. Mean nBV/TV was 62.47% (SD 19.46%), 51.01% (SD 24.43%) and 43.20% (SD 30.09%) in constructs with 2D BMSC, 3D BMSC and no cells, respectively (*p* > 0.05). Mean iBV/TV was generally low but greater in constructs with 3D BMSC (0.29%, range 0.03–0.96) versus 2D BMSC (0.08%, range 0–0.44; *p* > 0.05) and no cells (0.03%, range 0–0.07; *p* < 0.05) (Fig. 6b).

**Fig. 7:**
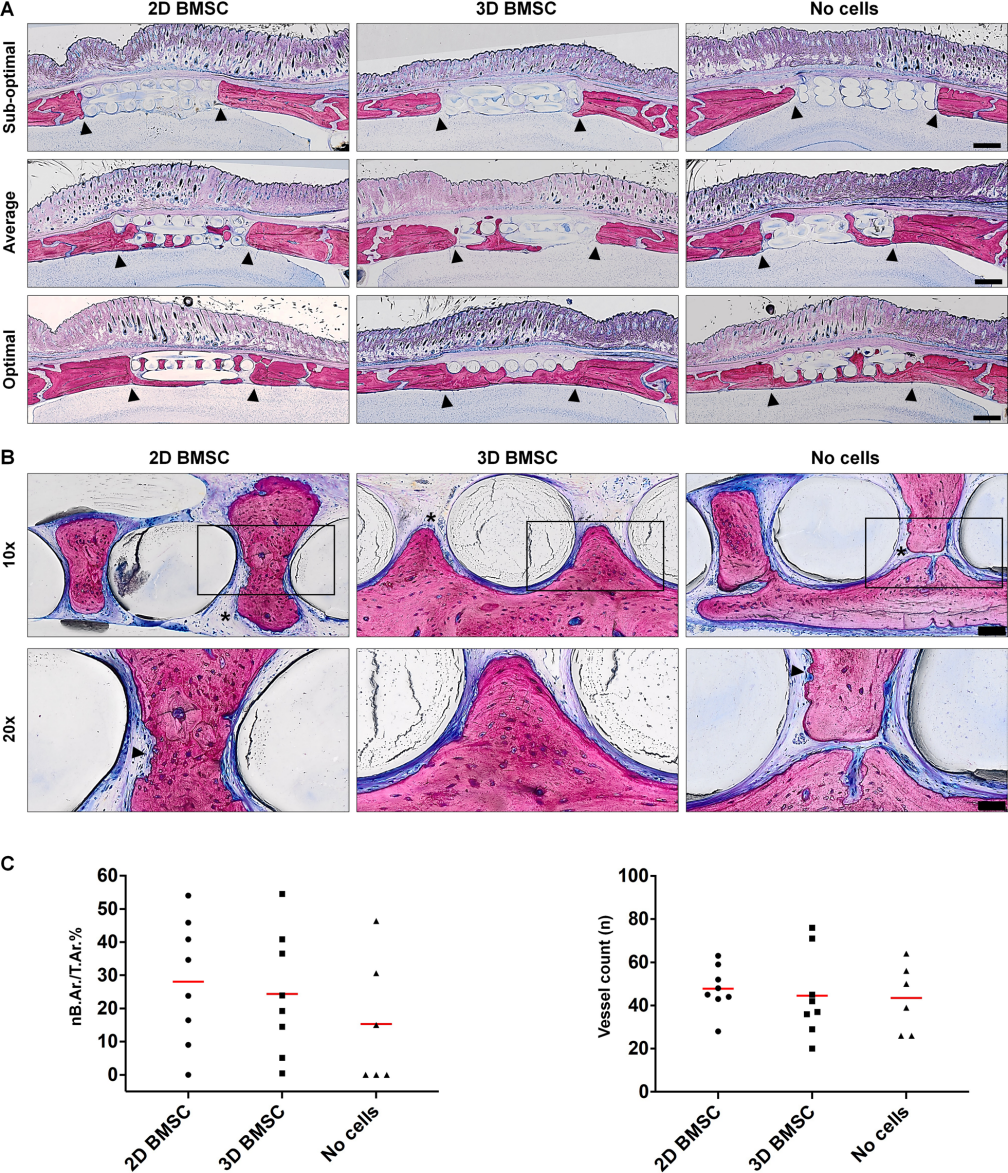
Histological analysis. **a** Representative low-magnification images from central sections of defects receiving constructs of 2D BMSC, 3D BMSC or no cells after 12 weeks, scale bars 1 mm; black arrows indicate original defect margins. Scalp and brain tissues are intact; mature bone appears light pink, woven bone dark pink and collagen dark blue. **b** Corresponding high-magnification images of newly formed bone, scale bars 100 µm (top panel) and 50 µm (bottom panel); black arrows indicate resorption lacunae suggestive of active remodeling. **c** Quantification of new bone formation (nB.Ar./T.Ar.%) and vessel counts (*n* = number/defect); data represent means

Morphology of the regenerated bone was evaluated via undecalcified histology of standardized sagittal sections in the centre of each defect. New bone mainly originated from the endocranial margins of the host calvarial bone and consisted predominantly of plexiform bone which is a combination of woven bone in the center and parallel-fibered bone on the superficial layers of bone trabeculae. This type of bone is formed during the initial stages of the healing of bone defects, and the process of primary bone formation was already completed at 12 weeks. No active osteoblasts or osteoblast seams were detectable on the trabecular surfaces (Fig. 7a). Blood vessels were strongly associated with areas of bone regeneration. The process of resorption of plexiform bone and replacement with lamellar bone, i.e., remodeling, could be observed via bone surfaces displaying resorption lacunae (Fig. 5b). No remarkable cellular inflammatory response was observed. A thin layer of fibrous tissue was always seen surrounding the scaffold and bone formation never seemed to occur directly on the scaffold surface (Fig. 7b). No visible signs of scaffold degradation were observed in any of the groups; scaffolds occupied ~ 50% of the defect area. Histomorphometry revealed a similar trend as the μCT analysis, with mean nB.Ar./T.Ar. of 28.09% (SD: 18.9%), 24.37% (SD: 18.49%) and 15.34% (SD: 19.51%) in constructs with 2D BMSC, 3D BMSC and no cells, respectively (*p* > 0.05, Fig. 7c). A similar degree of new vessel formation was observed in all groups (*p* > 0.05, Fig. 7c).

## Discussion

BTE is a promising strategy to treat advanced critical-size bone defects. In the present study, we compared the efficacy of PLATMC-HPLG constructs loaded with either dissociated (2D) BMSC, spheroid (3D) BMSC or no cells (cell-free controls) for bone regeneration in rat-calvarial defects. The main findings herein were (a) robust and comparable bone formation in constructs containing 2D or 3D BMSC and (b) favorable but non-significantly lower bone formation in cell-free PLATMC-HPLG constructs.

The efficacy of BMSC in BTE applications may be enhanced via xeno-free and spheroid culture. We recently reported the characterization of xeno-free spheroid cultures of BMSC in HPL [17]. Advantages of spheroid culture for multipotency, via upregulation of key regulator genes (BMP2, PPARG and SOX9), were confirmed herein. Consistent with previous results [17], upregulation of osteogenesis-related genes (BSP, OPN, OCN) was observed in 3D versus 2D BMSC herein, even in the absence of osteogenic supplements. Moreover, the secretion of several growth factors (FGF2, PDGF-BB, HGF, TGF-β2) and chemokines involved in tissue regeneration was also enhanced in 3D versus 2D BMSC. Thus, the two major mechanisms of MSC action, i.e., differentiation and paracrine function, appeared to be enhanced in 3D spheroids. Additionally, others have reported benefits of spheroid culture for MSC immunomodulatory functions in the context of tissue regeneration [21, 46, 47].

Although accumulating evidence suggests clear benefits of spheroid culture to enhance MSC efficacy, the optimal mode of spheroid delivery to regeneration sites has not been adequately investigated. Conventional strategies for in vivo delivery involve seeding of cells directly on scaffolds to allow attachment and spreading in vitro for a defined period prior to implantation. However, this method may not be optimal for delivering spheroids as it facilitates dissociation and migration of cells from the spheres during in vitro culture, thus compromising the benefits of cell aggregation. Interestingly, in one study, superior bone formation in rat-calvaria defects was observed when BMSC spheroids were transplanted as “suspensions” rather than when seeded on beta-tri-calcium phosphate (β-TCP) granules [22]. In contrast to direct seeding, encapsulation of spheroids in hydrogel scaffolds maintains their 3D assembly at the time of in vivo implantation. Recent studies have reported superior in vitro function and in vivo bone formation when using BMSC spheroids versus dissociated cells encapsulated in alginate hydrogels [29, 48, 49]. Since HPL was used as a xeno-free supplement for BMSC culture, its application was extended as a hydrogel carrier, via modification of previous methods [32]. Further, HPLG were supplemented with fibrin to improve their mechanical properties and prolong degradation, without compromising MSC function [28, 50–52].

In addition to HPLG, copolymer scaffolds were used to deliver the cells in vivo. Complex bone defects often necessitate the use of rigid biomaterial scaffolds, and in such cases hydrogels alone may be insufficient. 3D printing technology offers promising solutions for producing customized scaffolds to treat such defects. Although several designs and materials for 3D-printed scaffolds have been studied, their in vivo applications as carriers for human MSC have been limited [36]. PLATMC is reported to be a promising copolymer for various tissue engineering applications, particularly due to its mechanical properties and biocompatibility [37]; to our knowledge, no studies have yet tested its feasibility for BTE. Therefore, in the present study, human BMSC encapsulated in HPLG were combined with 3D-printed PLATMC scaffolds to represent the classical tissue engineering “triad” [53].

In a previous study we reported spontaneous upregulation of several osteogenesis-related genes in 3D versus 2D BMSC, regardless of osteogenic induction [13]. However, in the present study, no significant upregulation of RUNX2, ALP, COL1 or OCN was observed in 3D versus 2D BMSC following encapsulation in HPLG, regardless of osteogenic induction. This suggested that encapsulation in HPLG attenuated differences in gene expression between 2 and 3D BMSC. Nevertheless, a trend for superior in vitro mineralization was observed in encapsulated 3D versus 2D BMSC after 21 days of osteogenic induction. Considerable mineralization was also observed in non-induced constructs of 3D and 2D BMSC, suggesting a promotive effect of the HPLG on the osteogenic differentiation. Taken together, these findings suggest that the HPLG may have itself initiated the osteogenic differentiation of 2D and 3D BMSC (regardless of media supplements), thereby attenuating differences in gene expression, but promoting mineralization. Indeed, when used as a culture supplement, HPL promotes the osteogenic differentiation of MSC in vitro [54–56]. Moreover, several studies have reported positive effects of platelet-derived growth factors, e.g., PRP, on MSC osteogenic differentiation both in vitro and in vivo [23, 57–60]. Since HPL is being increasingly used for clinical-grade MSC expansion and may be easily and inexpensively produced using outdated platelet concentrates from blood establishments [10], HPLG represents a promising and cost-effective tool for BTE.

To test their potential for bone regeneration, PLATMC-HPLG constructs with 2D BMSC or 3D BMSC were implanted into rat-calvarial defects. In both groups, substantial bone regeneration could already be detected in the earliest in vivo CT scans after 4 weeks. After 12 weeks, robust bone regeneration was observed in both groups with maximum nBV/TV values of 91.5% and 87.56% and complete bridging in 6/8 and 5/8 defects in 2D and 3D BMSC constructs, respectively. Indeed, μCT analysis revealed a higher incidence of de novo bone island formation (iBV/TV) in 3D BMSC constructs, which could be attributed to in situ mineralization of the implanted spheroids with subsequent remodeling by host cells. However, since the formation of similar bone islands has also been reported in calvarial defects treated with only growth factors [44], i.e., without exogenous cells or scaffolds, the cellular origin of the bone islands remains elusive. In context, previous studies have reported enhanced regeneration in experimental bone defects treated with 3D versus 2D BMSC from allogeneic (rat) [25] or human sources [22, 29]. Similar outcomes were reported also in the context of periodontal ligament-derived cells (PDLC) in mouse calvarial defects [24]. Conversely, a recent study reported no differences in the healing of mouse femoral defects treated with either 2D or 3D human BMSC encapsulated in fibrin gels [61]. Consistent with this finding, no significant differences in the overall quantity or quality of regenerated bone were observed between 2 and 3D BMSC constructs in the present study. Notably, in the previous studies [22, 25, 29, 61], BMSC were cultured in OIM prior to implantation. Indeed, our in vitro data indicated superior mineralization in induced versus non-induced 2D and 3D BMSC constructs, despite some mineralization also being observed in non-induced constructs (“induction” in this context refers to the use of chemical stimulants such as dexamethasone, L-ascorbic acid and β-glycerophosphate, and not recombinant growth factors, such as BMP2). Nevertheless, for the in vivo experiments herein, non-induced constructs were used based on trends in recent clinical studies of BTE, and a preference for “minimal manipulation” of cells by regulatory authorities [1].

Although immunocompromised rodents are commonly reported animal models for testing human MSC [62], the precise mechanism(s) of bone formation in these animals, and the interactions between transplanted (human) and native (recipient) cells, have not been fully elucidated. In the present study, the histological technique (undecalcified) and lack of human-specific antibodies with low host-tissue cross-reactivity, precluded determination of the origin of newly formed bone tissues, i.e., whether these were formed by engraftment and differentiation of the transplanted human BMSC or via recruitment of host (rat) cells. Nevertheless, previous studies have reported that, depending on the immune status of experimental animals, transplanted BMSC may not differentiate into osteoblasts, but rather promote bone formation via paracrine stimulation of host cells [63–66]. In context, although significant differences were observed herein between the CM, i.e., paracrine effectors, of 2D and 3D BMSC, there were no differences in their ability to promote in vitro wound healing in rat BMSC (Additional file 1: Figure 3). Moreover, differences in cytokine secretions of 2D and 3D BMSC were not assessed following encapsulation in the constructs, which, like the differences in gene expression, may have been attenuated following encapsulation. Therefore, it may be speculated whether differences in paracrine functions between 2 and 3D BMSC (or a lack thereof), in addition to cross-species-related factors, contributed to the observed in vivo outcomes.

Comparatively lower, but favorable, regeneration was observed in cell-free (versus cell-loaded) PLATMC-HPLG constructs herein, with up to 84.9% nBV/TV (maximum) and bridging in 2/6 defects. This suggested (a) a possible stimulatory effect from HPLG on in vivo bone formation and (b) further supported the reports that osteogenesis mainly occurs via tissue-resident progenitor cells, and not via differentiation of the transplanted BMSC [63]. Indeed, HPL is known to contain a wide array of physiological growth factors which promote MSC differentiation in vitro [10]. In context, one study reported superior bone regeneration in calvarial defects when using 3D-printed PCL scaffolds coated with “freeze-dried PRP” versus uncoated scaffolds; optimal bone regeneration was observed when using PRP activated via freezing/thawing (similar to HPL) versus thrombin/calcium activation [67]. Indeed, the PLATMC scaffolds alone showed substantially lower bone regeneration herein, i.e., 9% and 15% defect fill, in two animals. Notably, no differences in bone regeneration were observed between PLATMC scaffolds with and without HPLG in these two animals (Additional file 1: Figure 4). Nevertheless, we cannot rule out a possible stimulatory effect of the hydrogel on bone regeneration in our experiments. Such a potentially confounding effect, together with a lack of osteogenic pre-induction of cells, and the presence of local physiological stimuli in the defect sites, may have masked differences between 2 and 3D BMSC in this orthotopic model.

In context of the in vivo outcomes herein, it must be acknowledged that the scaffold itself, although excluded from the μCT analysis, occupied a considerable volume of the defects and did not show any signs of degradation or replacement during the experimental period. PLATMC is reported to be a promising copolymer for various tissue engineering applications mainly due to its biocompatibility [35], although little is known regarding its in vivo degradation profile. Optimal properties of scaffolds for bone regeneration have been defined, such as an average pore size of 300–400 μm (with ≥ 50% total porosity) [68], and a degradation profile corresponding to the physiological rate of bone formation [69]. Although the recommendations for physical properties, i.e., pore size of ~ 400 μm and total porosity > 50%, were incorporated into the design of the scaffolds herein, no prior assessment of their degradation profile was performed. Our in vivo observations revealed that bone formation occurred around—but not in direct contact with, the printed filaments, i.e., the scaffolds were incorporated within but not replaced by the regenerated bone. However, no specific in vitro or in vivo assessment of scaffold degradation was performed herein. Moreover, no mechanical testing of the regenerated tissues was performed. Thus, longer-term outcomes such as in vivo degradation of PLATMC and the mechanical and biological function of these scaffold-bone “composite tissues,” require further investigation.

## Conclusions

Encapsulation of spheroid (3D) and dissociated (2D) BMSC in PLATMC-HPLG constructs attenuated the differences in osteogenic gene expression observed in standard 3D spheroid versus 2D monolayer cultures. Despite a non-significant trend for superior in vitro mineralization in constructs of 3D BMSC versus 2D BMSC, in vivo implantation revealed comparable bone regeneration between the groups in rat-calvarial defects. Interestingly, favorable but non-significantly lower bone regeneration was also observed in cell-free PLATMC-HPLG constructs. In summary, regardless of spheroid or monolayer cell culture, PLATMC-HPLG constructs represent promising scaffolds for BTE applications.

## Supplementary Information

**Additional file 1**. Supplementary methods.

## Data Availability

Additional data are included in Additional file 1 and can be made available by the authors upon request.

## Abbreviations

BTE: Bone tissue engineering
MSC: Mesenchymal stromal cells
BMSC: Bone marrow MSC
2D: Two-dimensional
3D: Three-dimensional
MCC: Mesenchymal cell condensations
HPL: Human platelet lysate
PLA: Poly-L-lactic acid
PGA: Polyglycolic acid
PCL: Polycaprolactone
PLGA: Poly-lactic-co-glycolic acid
TMC: Trimethylene carbonate
PLATMC: Poly-L-lactide-co-trimethylene carbonate
HPLG: HPL hydrogels
GM: Growth media
OIM: Osteogenic induction media
qPCR: Polymerase chain reaction
CM: Conditioned media
PBS: Phosphate-buffered saline
PRP: Platelet-rich plasma
CT: Computed tomography
VOI: Volume of interest
nBV/TV: New bone volume per total defect volume
μCT: Micro-CT
iBV/TV: Isolated bone volume per total defect volume
nB.Ar/T.Ar: Area of newly formed bone to the total available area
ANOVA: One-way analysis of variance
BMP2: Bone morphogenetic protein 2
PPARG: Peroxisome proliferator-activated receptor gamma
SOX9: SRY-box transcription factor 9
OCN/BGLAP: Osteocalcin
OPN/SPP1: Osteopontin
FGF2: Fibroblast growth factor
HGF: Hepatocyte growth factor
GCSF: Granulocyte colony-stimulating factor
TGF-β2: Transforming growth factor beta
CXCL1/GROα: Chemokine ligand 1
CXCL10: Chemokine ligand 10
CCL5: Chemokine ligand 5
SCF: Stem cell factor
VEGF: Vascular endothelial growth factor
SCGF-β: Stem cell growth factor beta
RUNX2: Runt-related transcription factor 2
ALP: Alkaline phosphatase
COL1: Collagen type 1
β-TCP: Beta-tri-calcium phosphate
PDLC: Periodontal ligament-derived cells
