# Supplementary material for: Bone regeneration in rat calvarial defects using dissociated or spheroid mesenchymal stromal cells in scaffold-hydrogel constructs
Source: Stem Cell Res Ther. 2021 Nov 14;12:575. doi: 10.1186/s13287-021-02642-w (PMC8591809; doi:10.1186/s13287-021-02642-w)
Supplement: Supplementary file 1 — Additional file 1. Supplementary methods. [file 13287_2021_2642_MOESM1_ESM.docx]

**Supplementary data**

***Supplementary tables***

**Supplementary table 1: Real time PCR primers**

| **Gene** | **TaqMan^®^ Assay ID** | **Amplicon length** |
| --- | --- | --- |
| **Housekeeping gene** |  |  |
| GAPDH | Hs 02758991_g1 | 93 |
| **Adipogenesis-related** |  |  |
| PPARG | Hs01115513_m1 | 90 |
| **Chondrogenesis-related** |  |  |
| SOX9 | Hs00165814_m1 | 102 |
| **Osteogenesis-related** |  |  |
| RUNX2 | Hs01047973_m1 | 86 |
| BMP2 | Hs00154192_m1 | 60 |
| ALPL | Hs01029144_m1 | 79 |
| COL1A2 | Hs00164099_m1 | 68 |
| BSP | Hs00195432_m1 | 67 |
| OPN (SPP1) | Hs00959010_m1 | 84 |
| OCN (BGLAP) | Hs01587814_g1 | 138 |

GAPDH glyceraldehyde 3-phosphate dehydrogenase, PPARG peroxisome proliferator activated receptor gamma, SOX9 sex determining region Y-box 9, RUNX2 runt-related transcription factor 2, BMP2 bone morphogenetic protein 2, ALPL alkaline phosphatase, COL1A2 Collagen type 1 alpha 2, BSP, bone sialoprotein, OPN/SPP1 Osteopontin, OCN/BGLAP Osteocalcin

**Supplementary table 2: Multiplex cytokine assay panel**

| **Abbreviation** | **Cytokine** |
| --- | --- |
| b-FGF/FGF2 | Basic fibroblast growth factor |
| G-CSF | Granulocyte colony stimulating factor |
| GRO-α/CXCL1 | Growth-regulated alpha protein/CXC ligand 1 |
| HGF | Hepatocyte growth factor |
| IP-10/CXCL10 | Interferon gamma-induced protein 10/CXC chemokine 10 |
| SCF/KITLG | Stem cell factor/KIT-ligand |
| SCGF-β | Stem cell growth factor |
| SDF-1α/CXCL12 | Stromal cell-derived factor 1 |
| PDGF-BB | Platelet-derived growth factor-BB |
| RANTES/CCL5 | Regulated on activation, normal T cell expressed and secreted |
| TGF-β1, 2 | Transforming growth factor-beta 1, 2 |
| VEGF | Vascular endothelial growth factor |
| MIF | Macrophage migration inhibitory factor |
| M-CSF | Macrophage colony-stimulating factor |

***Supplementary figures***

**Supplementary figure 1: Micro-CT-based planning of standardized histological sections**

**
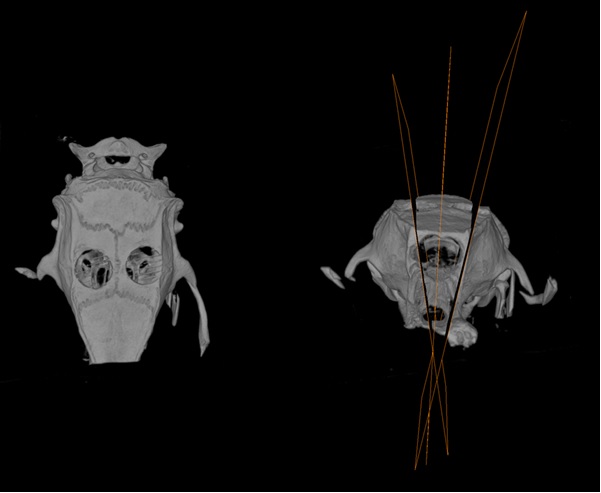
**

A virtual section was positioned in the μCT images through the centre of each defect in a plane parallel to the sagittal suture and perpendicular to the parietal bone. The section was then manually transferred to the resin blocks using reference points and measurements.

**Supplementary figure 2: Characterization of monolayer BMSC**

**B**

**A**

**
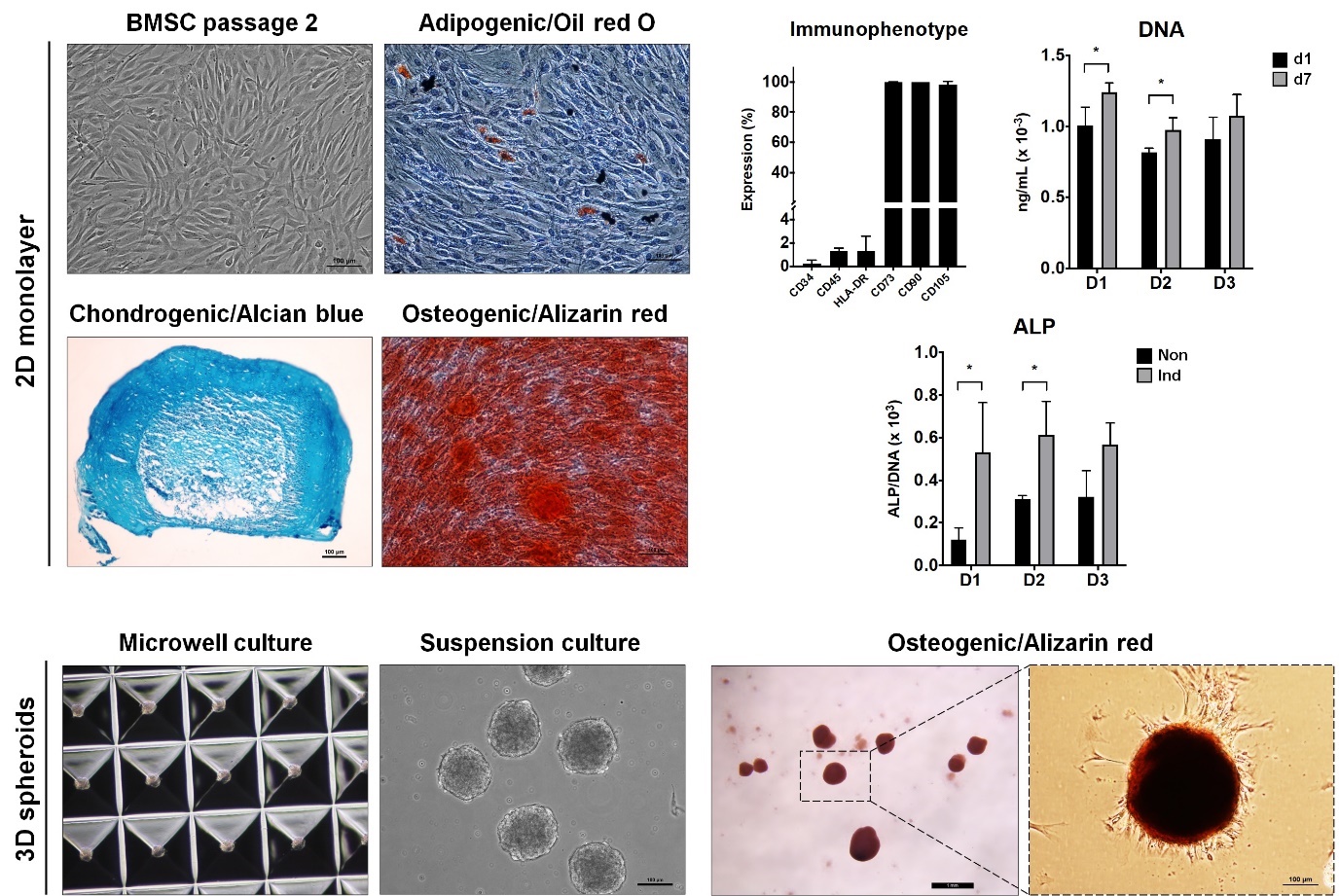
**

(A) Clockwise from left: representative images of monolayer BMSC morphology and multi-lineage differentiation along adipogenic, osteogenic and chondrogenic lineages; scale bars 100 um. (B) Clockwise from left: immunophenotype based on expression of surface markers via flow cytometry, n=3 donors; proliferation over 7 days via DNA quantification (n=3 donors); and alkaline phosphatase (ALP) assay in osteogenically induced (ind) and non-induced BMSC (n=3 donors); data represent means + SD, * *p* < 0.05; ** *p* < 0.001.

**Supplementary figure 3: In vitro wound healing assay**

**
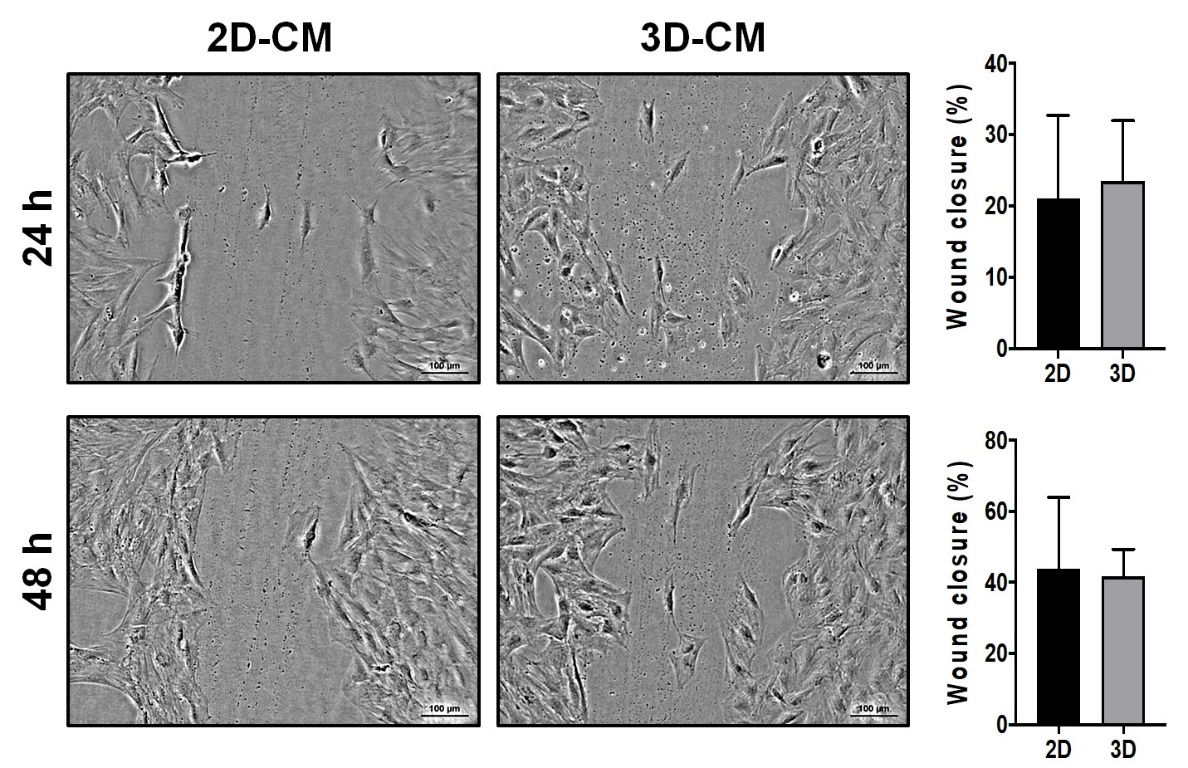
**

In vitro wound healing assay of rat BMSC showing the effect of CM from 2D (2D-CM) and 3D BMSC (3D-CM) after 24 and 48 h (scale bars 100 µm) and corresponding analyses of wound closure (n = >3 experimental replicates). No statistically significant differences between the groups.

**Supplementary figure 4: Calvarial bone regeneration in cell-free constructs**

**
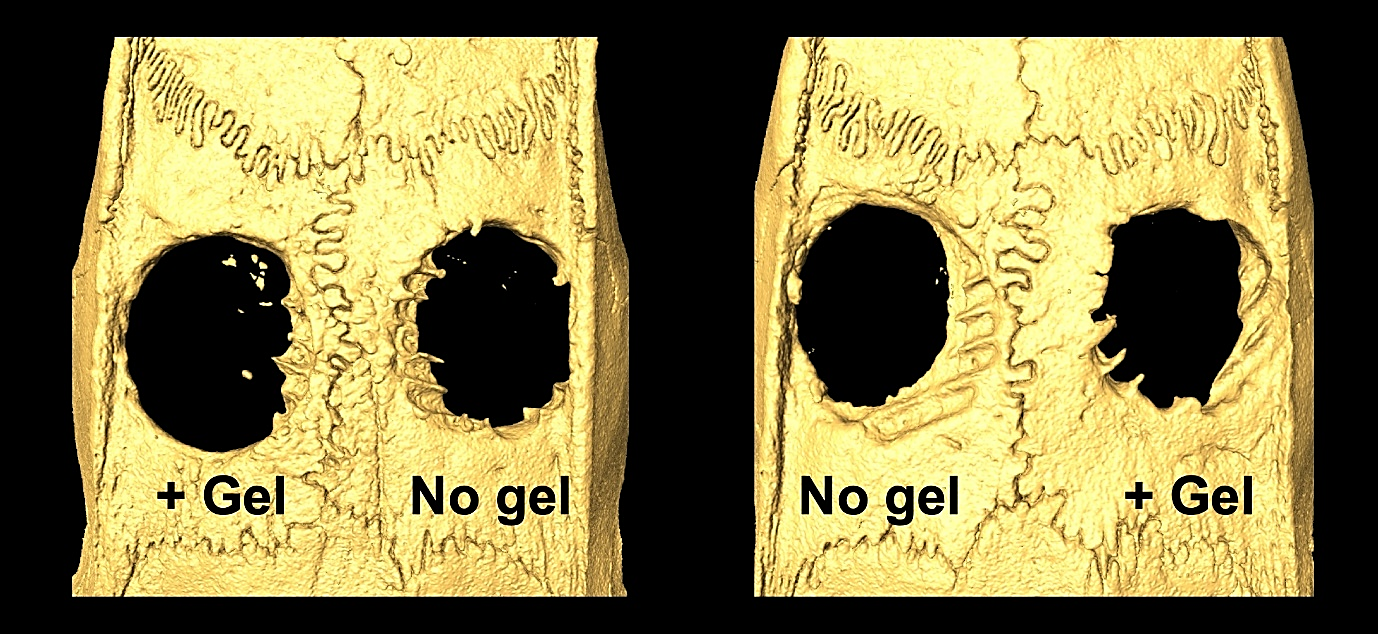
**

Micro-CT reconstructions of bone regeneration after 12 weeks in calvarial defects treated with PLA-TMC scaffolds alone (No gel, n=2) or in combination with HPL hydrogels (+ Gel, n=2).
